# Developmental disruption of perineuronal nets in the medial prefrontal cortex after maternal immune activation

**DOI:** 10.1038/srep37580

**Published:** 2016-11-23

**Authors:** John W. Paylor, Brittney R. Lins, Quentin Greba, Nicholas Moen, Reiner Silveira de Moraes, John G. Howland, Ian R. Winship

**Affiliations:** 1 Neuroscience and Mental Health Institute, University of Alberta, Edmonton, T6G 2E1, AB, Canada; 2 Neurochemical Research Unit, Department of Psychiatry, University of Alberta, Edmonton, T6G 2B7, AB, Canada; 3 Department of Physiology, University of Saskatchewan, Saskatoon, S7N 5E5, SK, Canada

## Abstract

Maternal infection during pregnancy increases the risk of offspring developing schizophrenia later in life. Similarly, animal models of maternal immune activation (MIA) induce behavioural and anatomical disturbances consistent with a schizophrenia-like phenotype in offspring. Notably, cognitive impairments in tasks dependent on the prefrontal cortex (PFC) are observed in humans with schizophrenia and in offspring after MIA during pregnancy. Recent studies of post-mortem tissue from individuals with schizophrenia revealed deficits in extracellular matrix structures called perineuronal nets (PNNs), particularly in PFC. Given these findings, we examined PNNs over the course of development in a well-characterized rat model of MIA using polyinosinic-polycytidylic acid (polyI:C). We found selective reductions of PNNs in the PFC of polyI:C offspring which did not manifest until early adulthood. These deficits were not associated with changes in parvalbumin cell density, but a decrease in the percentage of parvalbumin cells surrounded by a PNN. Developmental expression of PNNs was also significantly altered in the amygdala of polyI:C offspring. Our results indicate MIA causes region specific developmental abnormalities in PNNs in the PFC of offspring. These findings confirm the polyI:C model replicates neuropathological alterations associated with schizophrenia and may identify novel mechanisms for cognitive and emotional dysfunction in the disorder.

The etiology and underlying neurobiological mechanisms of schizophrenia (SCZ) are elusive despite the prevalence (1% population worldwide) and burden (1% global burden of disease) of the disorder^1^,^2^. Symptoms of SCZ typically fall into one of three clusters: positive (e.g., hallucinations and delusions), negative (e.g., social isolation, anhedonia), and cognitive (e.g., sensory processing dysfunction, impairments in working memory). Interestingly, while the symptoms of SCZ typically manifest during adolescence and early adulthood, a substantial body of research links prenatal events such as maternal infection during pregnancy to the risk for developing SCZ later in life^3^,^4^. As a result, animal models have been developed to further understand the relationship between prenatal infection and SCZ. Typically, these models involve inducing an immune reaction in pregnant rats or mice with agents such as the viral mimetic polyinosinic-polycytidylic acid (polyI:C). A growing literature supports the assertion that following maternal immune activation (MIA) during pregnancy, the offspring show behavioral, anatomical, neurochemical, and electrophysiological disturbances consistent with SCZ^5^,^6^,^7^,^8^,^9^,^10^,^11^,^12^,^13^,^14^,^15^.

In recent years, studies of post-mortem brain tissue from individuals with SCZ have revealed reduced densities of perineuronal nets (PNNs), highly specialized extracellular matrix structures closely associated with inhibitory interneurons^16^,^17^. PNNs surround the cell body, proximal dendrites, and initial axon segment of the neurons which host them, and are involved in a variety of processes which limit both structural and synaptic plasticity^18^,^19^,^20^,^21^,^22^,^23^,^24^,^25^,^26^. Deficits in PNNs are observed in the prefrontal cortex, entorhinal cortex, amygdala, and olfactory epithelium in post-mortem tissue from people with SCZ^27^,^28^,^29^,^30^,^31^. Interestingly, the expression of PNNs increases throughout postnatal development with the greatest increase during adolescence, a time corresponding with the onset for symptoms of SCZ in most patients^16^,^32^.

PNNs have well defined roles in the regulation of critical periods of brain circuit plasticity. Critical periods during neurodevelopment are characterized by dramatic structural and functional neuroplasticity in response to environmental stimuli or activity^24^,^33^. Most notably, increased PNN density is associated with the closure of critical periods during development of the visual cortex^24^. Malformation of PNNs may therefore dysregulate critical periods and contribute to heightened synaptic pruning^17^,^34^,^35^. Moreover, PNNs are closely associated with parvalbumin expressing inhibitory interneurons (PV+), which are also intimately involved in regulation of critical periods in cortex^35^. Mature PV+ interneurons are fast-spiking inhibitory interneurons involved in regulating network activity, particularly with respect to the generation of gamma oscillatory activity^36^,^37^,^38^. Notably, PV cells and gamma oscillations are altered in SCZ^39^,^40^,^41^,^42^,^43^,^44^,^45^. PNNs support the high metabolic demand of these interneurons and contribute to ion homeostasis around them^19^,^20^,^46^,^47^. However, the functional relationship between PNNs and PV+ interneurons in the development of SCZ is still unknown.

While recent evidence suggests that PNNs and PV interneurons are selectively disrupted in SCZ, post-mortem studies are limited by their small sample sizes, limited demographics, the chronic use of psychiatric medications, and heterogeneous comorbidities. In addition, post-mortem studies do not allow for assessment of the cellular and molecular changes during development that may precede the onset of symptoms in SCZ. Therefore, the present study was designed to define abnormalities in the density of PNNs and PV+ cells in brain regions implicated in SCZ in the offspring of rats subjected to MIA using the viral mimetic polyI:C. In previous studies from our group, the behavioral phenotype of MIA rat offspring has been characterized. Young adult offspring (approximately PND 56–98) of polyI:C-treated dams display disrupted prepulse inhibition, locomotor activity, behavioral flexibility, recognition memory, crossmodal object memory, and exhibit impaired regulation of fear behavior^10^,^12^,^13^,^14^. Therefore, we chose to examine the expression of PNN in the offspring of rat dams treated in the same manner as these previous studies. Our results reveal age- and brain region-dependent disruptions of PNNs and PV cells, providing insight into the neurobiology underlying cognitive and social dysfunction associated with SCZ.

## Results

### Effects of polyI:C treatment on the dams and litters

**Figure 1:**
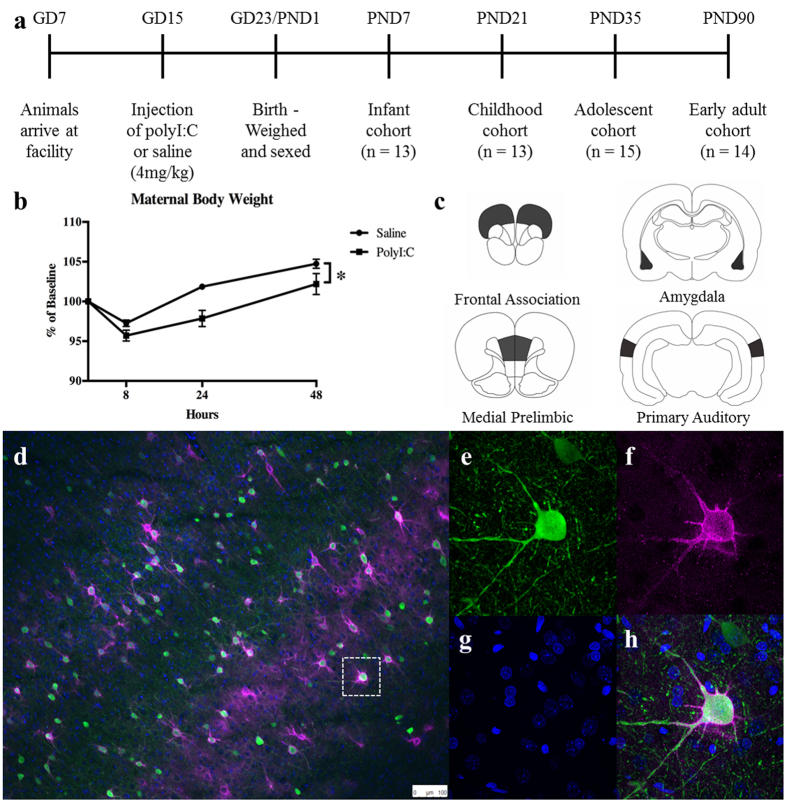
(**a**) Experimental timeline and setup. See Methods for details. (**b**) Dams receiving injections of polyI:C had significant weight loss compared to saline-injection dams over the 48 hours after injection (main effect of time, *p* < 0.0001; treatment, *p* < 0.05). (**c**) Regions of interest for our analysis. (**d**) Representative examples of PV cells (green), DAPI nuclei (blue) and PNNs, stained with wisteria floribunda agglutinin (WFA; purple), in the cortex. (**e–h**) An example high resolution image of a single PV cell surrounded by a PNN.

Dams received a single injection of either polyI:C or saline on GD15 consistent with protocols used previously^10^,^12^,^13^,^14^ (Fig. 1a). Administration of polyI:C to the pregnant dams induced a significant reduction in weight gain (Fig. 1b). Analysis of the weight of the dams (8, 24, and 48 h after injection) confirmed a significant main effect of time (F(2,30) = 98.52, p < 0.0001) and a significant main effect of treatment (F(1,15) = 5.30, p < 0.05; interaction p > 0.10). PolyI:C treatment did not affect maternal temperature (data not shown), consistent with previous studies from our group^10^,^12^,^13^,^14^. Average litter size (saline = 11.43 ± 0.6 pups/litter; polyI:C = 11.60 ± 1.2 pups/litter) and average pup weight at birth (saline = 7.04 ± 0.28 g/pup; polyI:C = 7.58 ± 0.27 pups/litter) were also not altered by treatment (statistics not shown).

### Effects of polyI:C treatment on PNNs and associated markers

Data were collected from four brain regions (Fig. 1c; frontal association cortex, medial prelimbic cortex, basolateral nucleus of the amygdala, and primary auditory cortex) at four developmental ages (PND7, 21, 35, and 90). For each region, we measured total PNN count, PV+ cell density, IBA1+ cell density, GFAP staining intensity and DAPI+ nuclei density (examples shown Fig. 1d–h).

### Medial Prelimbic Cortex

**Figure 2:**
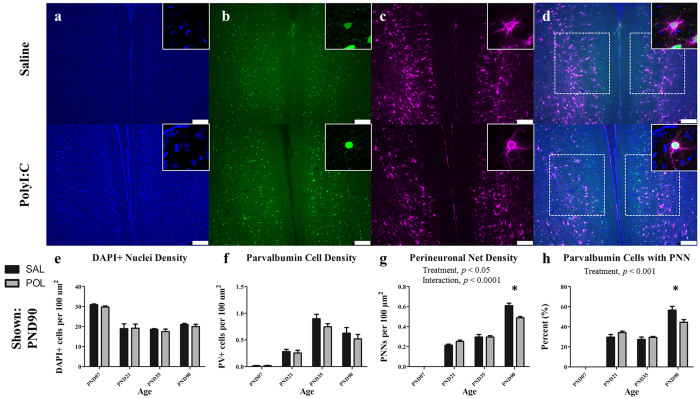
The medial prelimbic cortex has deficits in PNNs that emerge during early adulthood (PND90). Panels show DAPI (**a**), PV+ cells (**b**), PNNs ((**c**); stained with WFA) from representative rats at PND90. Across both conditions the total number of DAPI labelled nuclei decreased from PND7 to PND21 before plateauing (main effect of Age, *p* < 0.0001). PV cell density increased from PND7 to PND35 before declining at PND90 (main effect of Age, *p* < 0.0001). PNN density increased throughout postnatal development with the greatest increases occurring from PND7 to 21, and PND35 to 90 (main effect of Age, *p* < 0.0001; Age × Treatment, *p* < 0.0001; Treatment, *p* < 0.05). In the PND90 cohort, a significant deficit in PNN density emerged in polyI:C treated animals as compared to saline-treated (*p* < 0.0001). There was also a significant reduction in the number of PV cells ensheathed in a PNN (main effect of Age, p < 0.0001; Treatment, p < 0.001). Insets are representative images from each condition. Scale bar represents 250 μm. *p<0.001.

Representative immunofluorescent images from medial prelimbic cortex from an animal at PND 90 are illustrated in Fig. 2 (a–c show DAPI, PV, and WFA labeling, respectively, d shows a merged image). Consistent with previous research in human post-mortem tissue, PNNs were absent in infancy and increased throughout postnatal development in the prefrontal cortex^30^ (Fig. 2g). A two-way ANOVA revealed a significant overall effect of age on PNN density, (*F*(3,47) = 479.10, *p* < 0.0001), a significant main effect of prenatal treatment with PolyI:C (*F*(1,47) = 4.12, *p* < 0.05) and a significant interaction between treatment and age (*F*(3,47) = 11.20, *p* < 0.001). Post-hoc comparisons revealed a significant decrease in PNN density within the medial prelimbic cortex of polyI:C treated offspring relative to offspring from saline-treated dams at PND90 [SAL (*M* = 0.60 ± 0.03) vs. POL (*M* = 0.49 ± 0.01), *t* = 5.84, *p* < 0.0001]. As PNNs are closely associated with PV interneurons, we next evaluated PV cell density within the same region (Fig. 2f). A two-way ANOVA on PV neuron density revealed a significant overall effect of age, (*F*(3,47) = 56.59, *p* < 0.0001) but no significant main effect of treatment or age X treatment interaction. An analysis of the percentage of PV cells ensheathed by PNNs revealed a significant main effect of age (Fig. 2h) (*F*(3,47) = 203.3, *p* < 0.0001) and a significant interaction between treatment and age (*F*(3,47) = 6.36, *p* < 0.01). Post-hoc comparisons revealed a significant reduction in the percentage of PV cells surrounded by a PNN in polyI:C treated animals in the PND90 cohort relative to saline controls [SAL (*M* = 56.62 ± 3.72) vs. POL (*M* = 44.54 ± 0.70), *t* = 4.12, *p* < 0.001]. A two-way ANOVA on overall (DAPI+) cell density in prelimbic cortex revealed a significant effect of age (Fig. 2e) (*F*(3,47) = 31.28, *p* < 0.0001) but no significant effect of polyI:C treatment or interaction between treatment and age.

### Frontal Association Cortex

**Figure 3:**
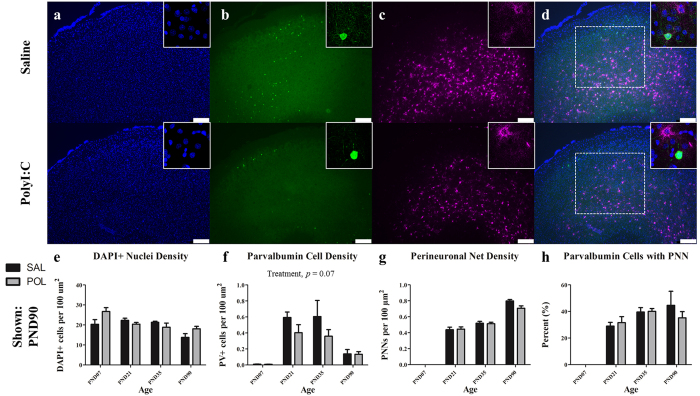
The frontal association cortex has developmental disturbances in PV+ cell density. Panels show DAPI (**a**), PV+ cells (**b**), PNNs ((**c**); stained with WFA) from representative rats at PND90. Across both conditions the total number of DAPI labelled nuclei decreased steadily from infancy to early adulthood (main effect of Age, *p* < 0.0001). PV+ interneurons increased from PND7 to PND35 before declining at PND90 (main effect of Age, *p* < 0.0001). While PV+ cell density was reduced across development in polyI:C treated animals, this effect was not significant (main effect of Treatment, *p* = 0.07). The number of perineuronal nets increased throughout postnatal development (main effect of Age, *p* < 0.0001). Insets are representative images from each condition. Scale bar represents 250 μm. *p < 0.001.

Representative immunofluorescent images of frontal association cortex from an animal at PND 90 are illustrated in Fig. 3. PNN density within the frontal association cortex increased throughout postnatal development into adulthood (Fig. 3g; *F*(3,47) = 340.8, *p* < 0.0001), but there was no significant effect of treatment or an interaction between treatment and age. PV cell density varied with age (Fig. 3f; *F*(3,47) = 17.44, *p* < 0.0001) but the effect of treatment on PV cell density did not reach statistical significance (*F*(1,47) = 3.52, *p* = 0.07; age X treatment interaction *p* > 0.10). A two-way ANOVA on percentage of PV cells ensheathed by a PNN revealed a significant effect of age (Fig. 3h; *F*(3,47) = 32.90, *p* < 0.0001), but no effect of treatment nor an interaction between treatment and age. DAPI+ cell density varied with age (Fig. 3e; *F*(3,47) = 6.44, *p* < 0.01). There was no effect of polyI:C treatment on DAPI+ cell density but a significant interaction between treatment and age was detected (*F*(3,47) = 3.11, *p* < 0.05).

### Amygdala

**Figure 4:**
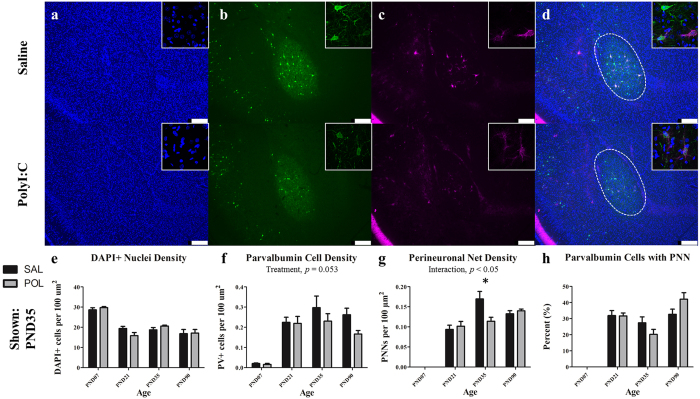
The amygdala has deficits in PNNs that emerge during adolescence (PND35). Panels show DAPI (**a**), PV+ cells (**b**), PNNs ((**c**); stained with WFA) from representative rats at PND90. Across both conditions the total number of DAPI labelled nuclei decreased from infancy to childhood before plateauing (main effect of Age, *p* < 0.0001). PV+ interneurons increased from PND7 to PND35 before declining at PND90 (main effect of Age, *p* < 0.0001). There was an effect of reduced PV cell density across development, however this did not reach statistical significance (main effect of Treatment, *p* = 0.053). The number of perineuronal nets increased throughout postnatal development until PND35 (main effect of Age, *p* < 0.0001; Age × Treatment, *p* < 0.01). Within the PND35 cohort, there was a significant reduction in PNN density in polyI:C treated animals (*p* < 0.001). The percentage of PV cells surrounded by a PNN varied throughout development by cohort (main effect of Age, *p < *0.0001). Insets are representative images from each condition. Scale bar represents 250 μm. *p < 0.001.

Representative immunofluorescent images from the basolateral amygdala from an animal at PND 35 are illustrated in Fig. 4. A two-way ANOVA on PNN density within the basolateral amygdala revealed a significant overall effect of age (Fig. 4g; *F*(3,45) = 109.1, *p* < 0.0001) and a significant interaction between treatment and age (*F*(3,45) = 5.27, *p* < 0.005). Post-hoc comparisons revealed a significant reduction in PNN density in polyI:C treated offspring at PND35 [SAL (*M* = 0.17 ± 0.02) vs. POL (*M* = 0.11 ± 0.01), *t* = 3.17, *p* < 0.05]. Analysis of PV cell density within the basolateral amygdala revealed a significant effect of age (Fig. 2f; *F*(3,45) = 27.28, *p* < 0.0001). A main effect of treatment on PV density approached statistical significance (*F*(1,45) = 3.947, *p* = 0.053, interaction *p* > 0.10). A two-way ANOVA evaluated on percentage of PV cells with a PNN also revealed a significant effect of age, (Fig. 2h; *F*(3,45) = 62.38, *p* < 0.0001). A main effect of treatment on PV cells with a PNN did not reach statistical significance (*F*(3,45) = 2.39, *p* = 0.081). A two-way ANOVA on DAPI+ cell density indicated a significant effect of age (*F*(3,45) = 42.37, *p* < 0.0001) only.

### Primary Auditory Cortex

Within the primary auditory cortex, PNN density, PV cell density, percentage of PV cells ensheathed by a PNN, and overall DAPI+ nuclei density varied significantly with age (PNNs, *F*(3,46) = 330.90, *p* < 0.0001; PV cell density, *F*(3,46) = 57.90, *p* < 0.0001; PV cells with PNN, *F*(3,46) = 90.18, *p* < 0.0001; DAPI+ cell density, *F*(1,46) = 68.84, *p* < 0.0001). There was no effect of polyI:C treatment or significant interaction between age and treatment for any of these measures.

### Immune Cells

**Figure 5:**
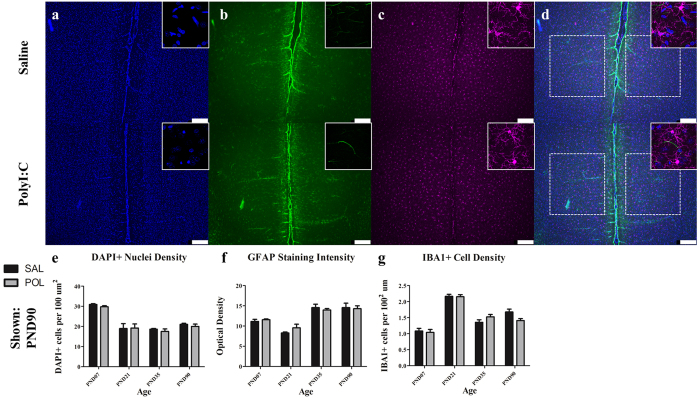
The medial prelimbic cortex exhibited no overt signs of microglial or astrocytic reactivity (PND90 shown). Panels show DAPI (**a**), IBA1 (**c**) and GFAP (**b**). Across both conditions the total number of DAPI labelled nuclei decreased throughout postnatal development (main effect of Age, *p* < 0.0001). Microglial density was increased slightly throughout postnatal development aside from a dramatic spike in density in the PND21 cohort (main effect of Age, *p* < 0.001; Age × Treatment, *p* = 0.056). GFAP staining was sparsely present within the cortical parenchyma, instead clustering around the cortical surface and blood vessels throughout the tissue. GFAP staining intensity increased from PND21 to PND35 where it leveled off into maturity (main effect of Age, *p* < 0.0001). Neither polyI:C treated animals or controls showed significant differences between either of these immune markers. Insets are representative images from each condition. Scale bar represents 250 μm. *p < 0.05.

We evaluated microglial cell density all four brain regions in each developmental cohort (Fig. 5). In the PND7 cohorts from both treatment groups, IBA1-labelled microglia appeared to display a more spherical morphology as compared to the ramified appearance of later developmental cohorts. Across all regions, we did not observe any statistically significant effects of treatment on microglial density, but there were significant age effects. For example, in the medial prelimbic cortex microglial cell density varied with age (Fig. 5e; *F*(3.47) = 65.57, *p* < 0.0001). There was no effect of treatment and the interaction between age and treatment on microglial cell density did not reach statistical significance (Fig. 5g; *F*(3,47) = 2.69, *p* = 0.057, treatment *p* > 0.10). We also assessed astrocytes by measuring total GFAP optical density within target regions as well as by qualitative observation. Analysis of GFAP staining intensity within the medial prelimbic cortex revealed a significant main effect of age (Fig. 5f; *F*(3,47) = 31.83, *p* < 0.0001). GFAP staining was largely absent in the PND7 cohort and in most regions increased throughout postnatal development. In adults, GFAP was localized primary to layer 1 of the cortex and around apparent blood vessels. We did not observe statistically significant changes in GFAP optical density or apparent qualitative changes in between treatment groups in any brain region.

## Discussion

In the present study, we showed that PNNs are reduced in the medial prefrontal cortex of adult rats treated prenatally with polyI:C. These deficits are not accompanied by overall reductions in the density of PV+ interneurons, but instead reflect a reduction in the percentage of PV+ cells surrounded by a PNN in prelimbic cortex. Reduced PNNs were also observed at some developmental ages in the basolateral amygdala. Developmental reductions of PV+ cell density in the basolateral amygdala and frontal association cortex did not reach statistical significance. There were no changes to PNNs or PV cell density within the primary auditory cortex, and we did not observe any overt indications of reactivity or proliferation of astrocytes or microglia in offspring after prenatal treatment with polyI:C.

To our knowledge, our study is the first to describe developmental abnormalities in PNN density and PV cell ensheathment in the polyI:C model and the first to report PNN deficits in the medial PFC of affected animals in early adulthood. These findings are consistent with several studies investigating post-mortem tissue of schizophrenic patients which have identified PNN deficits in the PFC^30^,^31^. Excitingly, our findings suggest PNN loss is coincident with age-of-onset of cognitive symptoms in the MIA model. In contrast, post-mortem tissue from human SCZ patients can typically only be obtained long after a diagnosis and PNN integrity could be influenced by many confounding factors. Notably, PNNs are closely associated with PV+ interneurons which have been extensively studied in SCZ and are likely central to dysfunction of cortical inhibition in the disorder^48^,^49^,^50^. While we did not find reductions in PV+ interneuron density within the prelimbic cortex, we did observe reductions in the percentage of PV+ interneurons surrounded by a PNN. Studies in human post-mortem tissue have identified stable PV+ cell densities within the medial PFC, but reduced expression of PV+ protein and mRNA within these cells, as well as reduced GAD67 expression^39^,^42^,^44^,^50^,^51^,^52^. Similarly, studies utilizing the polyI:C model have described changes to GAD65 and -67 within the prelimbic cortex in affected animals. Richetto and colleagues (2014) found evidence of decrease GAD65 and -67 mRNA and protein levels within the prelimbic cortex that did not emerge until PND95–106^53^. Notably, Cassella and colleagues (2016) described GAD67 mRNA reductions in the prelimbic cortex at PND60 that were not present at PND30^54^. It has also recently been reported that there are functional disturbances to PV+ interneurons in the medial PFC as a result of prenatal polyI:C treatment^55^. Therefore, the loss of PNNs reported here is consistent with other disturbances to PV+ interneurons occurring during adolescence and early adulthood within the medial PFC of polyI:C offspring.

The medial PFC is implicated in cognitive functions related to schizophrenia, including working memory, attentional control, sensorimotor gating, and behavioural flexibility^56^,^57^,^58^. PolyI:C affected offspring have deficits in tests of these constructs, including object recognition tasks of working memory, set-shifting tasks of behavioural flexibility, and prepulse inhibition of sensorimotor gating^10^,^12^,^59^,^60^,^61^,^62^. Importantly, our group and others have shown that these behavioural deficits do not emerge until adolescence and early adulthood (PND 56–98), the same window within which PNN deficits in the medial prelimbic cortex were observed in this study. However, it should be noted that alterations in some other measures such as ultrasonic vocalizations have been detected in polyI:C mouse offspring during the early postnatal period^63^. PNNs contribute to stabilizing neuronal structure, restricting neuronal plasticity, and supporting the physiological demands of host neurons^64^,^65^,^66^. Disruption of this supportive matrix could destabilize neuronal connectivity and dysregulate their physiological activity, though this remains to be demonstrated experimentally. Further study into the functional and structural consequences of PV+ cells losing their PNNs would clarify the contribution of PNN deficits to the behavioral changes in the polyI:C model and ultimately schizophrenia.

Post-mortem studies from SCZ patients have identified deficits in PNNs within the amygdala^27^. Similarly, our data identify deficits in PNN density in the basolateral amygdala, but only within our adolescent cohort and not later in adulthood. While the transiency of this deficit is difficult to interpret, adolescence is a particularly critical time for development of the amygdala, especially with regard to fear memories and extinction phenotypes^67^. PolyI:C affected animals are known to have a variety of deficits in fear processing, however many of these do not emerge until later adolescence and early adulthood^13^,^62^,^68^. Developmental reductions in PV+ cell density in the amygdala and the frontal association cortex did not reach statistical significance. In the human brain structure analogous to the frontal association cortex, the anterior prefrontal cortex (Brodmann Area 10), reduced PV+ cell density has been reported in SCZ patient’s brains post-mortem^69^. In contrast, previous studies that identified PNN deficits in the amygdala have found stable PV+ cell densities^27^. The high correlation between effects observed here in the basolateral amygdala and frontal association are intriguing considering the reciprocal connectivity between these two structures that are integral to associative learning, particularly with respect to fear learning and memory^70^.

Preliminary examination of microglia and astrocytes did not indicate ongoing inflammatory reactivity or proliferation in the brains of polyI:C treated offspring compared to controls. In addition to neurons themselves, astrocytes are major secretors of PNN components including chondroitin sulfate proteoglycans^71^,^72^. Consistent with this, astrocytic staining was sparse during infancy but increased throughout development, similar to the formation of PNNs. While astrocytes secrete ECM components, microglia are the primary secretors of the enzymes which degrade the ECM^73^,^74^. These include enzymes like MMPs (matrix metalloproteinases) and ADAMTSs (a disintegrin and metalloproteinase with a thrombospondin motif). However, we observed no signs of changes in microglial density or gross morphology, consistent with other studies that examined microglia after prenatal polyI:C treatment^75^,^76^. The lack of ongoing inflammatory processes suggests that the effects we observed are the consequence of disturbed developmental trajectories rather than ongoing immune dysfunction as a result of prenatal infection. However, it should be noted that cell density alone is not a sensitive measure to the full scope of microglia’s role in CNS maintenance. It is possible that the functional attributes and secretion of ECM degrading enzymes by microglia could be dramatically altered without changes in their density. It has previously been shown that MMP-9 is elevated in the brains of SCZ patients and mutations in the gene associated with MMP-16 were recently identified to confer heightened SCZ risk in a genome-wide association scan^77^,^78^,^79^,^80^. Thus, the relationship between microglia and the ECM, PNNs in particular, remains an intriguing avenue for future research.

These data demonstrate that the polyI:C model shares the PNN disturbances in the medial PFC observed in SCZ patients brains post-mortem. Importantly, these deficits do not emerge until during adolescence and early adulthood, matching the age of onset for SCZ-like symptoms in polyI:C animals and humans with SCZ. This pathology adds further validity to the MIA model of SCZ, and identifies a neurodevelopmental PNN deficit in prefrontal cortex as a potential contributor to the disabling cognitive impairment associated with SCZ. While changes in PNNs and PV+ cells have been reported in models and humans, the functional significance of these changes remain to be delineated. A better understanding of PNNs functional role in the prefrontal cortex and the means by which they are maintained or disturbed will facilitate the potential development of therapeutic strategies to prevent or compensate for their loss in SCZ.

## Methods

### Subjects and Housing

Timed-pregnant Long-Evans rats (n = 17; Charles River Laboratory, Quebec, Canada) arrived at the vivarium on gestational day (GD) 7 and were single housed in clear, ventilated plastic cages (Fig. 1a). Lighting was controlled automatically on a 12:12 hour cycle with lights on at 7:00 am. All handling and experimentation occurred within the light phase. Food (Purina Rat Chow) and water were available *ad libitum*. All experiments were performed in accordance with the standards of the Canadian Council on Animal Care and were approved by the University of Saskatchewan Animal Research Ethics Board.

### Treatment Procedure

Treatment of the dams was according to the well-established protocol from Howland’s group used in previous studies of behavioural abnormalities^10^,^12^,^13^,^14^. On GD 15, the dams were weighed and rectal temperature were recorded (Homeothermic Blanket System, Harvard Instruments, MA, USA). Dams were then anesthetised for approximately 6 minutes using isoflurane (5% induction, 3% maintenance) and administered a single tail vein injection of either saline (n = 7) or polyI:C (4 mg/kg, high molecular weight; InVivoGen, San Diego, CA, USA; n = 10).

### Follow-up Care of Dams and Pups

Body weight and rectal temperature measurements were taken again from the dams 8, 24, and 48 h after treatment and the rats were then left undisturbed to give birth naturally. On postnatal day (PND) 1, pups were weighed, sexed, and culled to a maximum of 10 per litter (6 males where possible). Cages were changed twice per week and otherwise the litters were left undisturbed. Weaning occurred at PND 21 when pups were placed in same-sex sibling groups of 2 or 3. Standard care was provided until tissue collection. We have shown previously that this treatment protocol induces increased proinflammatory cytokines in the dams and replicable maternal weight loss in the polyI:C-treated dams^10^,^12^,^13^,^80^.

### Tissue Collection

On PND 7 (n = 13; 6 saline pups from 5 litters; 7 poly pups from 4 litters), 21 (n = 13; 6 saline pups from 4 litters; 7 poly pups from 7 litters), 35 (n = 15; 5 saline pups from 4 litters; 10 poly pups from 6 litters), or 90 (n = 14; 5 saline pups from 5 litters; 9 poly pups from 9 litters), pups were deeply anesthetised with isoflurane and perfused transcardially with saline followed by 4% paraformaldehyde using infusion pumps. Flow rate and volume of perfusate were adjusted based on pup size. Following perfusion, the brains were carefully extracted and stored in 4% paraformaldehyde at 4 °C. Twenty four h later, the brains were transferred to a 30% sucrose and 0.1% sodium azide solution for several days and flash frozen in isopentane. Frozen brains were then mounted and sectioned (25 um) on a cryostat.

### Immunohistochemistry

Slides were left to thaw to room temperature for 20 min and given three washes in 1X PBS for 10 min each. They were then incubated for 1 h with 10% Protein Block, Serum-Free (Dako, Mississauga, ON) in 1X PBS. After this, slides were incubated overnight at room temperature with a primary antibody in an antibody solution of 1% Protein Block, 1% Bovine Serum Albumin and 98% 1X PBS with 0.1% Triton X-100. The following primary antibodies were used: Wisteria Floribunda Agglutinin (WFA; 1:1000; Vector Labs, Philadelphia, PA), mouse anti-Parvalbumin (1:2000; Swant, Switzerland), rabbit anti-IBA1 (1:200; Dako, Mississauga, ON); and mouse anti-GFAP (1:200; Sigma-Aldrich, Oakville, ON). Slides were washed again three times, twice in 1X PBS with 1% tween-20 and then once in 1X PBS. Sections were then incubated for 1 h at room temperature with secondary antibodies in antibody solution. Secondary antibodies were as follows: Streptavidin 647 (1:200; Invitrogen, Burlington, ON), donkey anti-mouse Alexa Fluor® 488 (1:200; Molecular Probes, Eugene, OR) and donkey anti-rabbit Alexa Fluor® 647 (1:200; Molecular Probes, Eugene, OR). Slides were again washed three times, twice in PBS with 1% Tween-20 and once in 1X PBS. Slides were mounted with DAPI (4′,6-diamidino-2-phenylindole in vectashield mounting medium).

### Imaging

Images were captured on a Leica DMI6000B Microscope and processed with LAS AF computer software. All images were captured at 5X magnification over target regions, with a total of 4–6 images taken bilaterally in adjacent sections. Within each region and each cohort a constant gain, exposure, and light intensity were used for all images. Images for insets were captured using a Leica DMI4000 confocal microscope. All confocal images were captured with a 40X objective lens and within each region constant imaging parameters were used for all images.

### Identification of Brain Regions

All brain regions were identified using The Rat Brain in Stereotaxic Coordinates^81^ and selected based on their DAPI nuclear staining pattern (Fig. 1c). The frontal association cortex (region FrA) was identified between +4.70 mm to +5.20 mm anterior to Bregma with the imaging area adjusted to the dorsal edge of the slice extending through cortical layers 1–6. The prelimbic cortex (region PrL) was identified between +3.20 mm to +3.70 mm where the imaging area was centered over the anterior longitudinal sulcus capturing the all layers of the prelimbic cortex bilaterally in a single image. The basolateral nucleus of the amygdala (region BLA) was identified between −2.12 mm and −2.80 mm based on its teardrop nuclear staining pattern which is also readily identifiable by its parvalbumin staining distribution. The primary auditory cortex (region Au1) was identified between −4.16 mm and −5.20 mm and selected from the most lateral point of the cortical surface extending upwards

### Quantification

Quantification was completed on unmodified images by an observer blind to the specific experimental conditions of tissue analyzed. Cell counts for DAPI+, IBA+, and PV+ cells were all counted using the Image-based Tool for Counting Nuclei (Centre for Bio-image Informatics, UC Santa Barbara, CA, USA) plugin for NIH ImageJ software. Within a target region, a standard total area was measured over the region of interest within which cells were identified and cell counting parameters kept constant. PNNs were counted manually using the ImageJ Cell Counter function within a standard total area over the target region. For each stain and each region, measurements of mean brightness within the area were also taken. Measurements and counts for each brain region are the average of 2 images taken bilaterally from 2 adjacent sections.

### Statistical Analyses

All data are presented as mean ± SEM. Statistical analyses were carried out in PRISM Software (Prism Software, Irvine, CA, USA) using two-way ANOVAs with Bonferroni post hoc tests. Significance was set at *P* < 0.05. *P*-values < 0.10 are reported in the results section. Both sexes were included for analyses but comparisons between sexes were not done as several cohorts had limited numbers of female offspring.

## Additional Information

**How to cite this article**: Paylor, J. W. *et al*. Developmental disruption of perineuronal nets in the medial prefrontal cortex after maternal immune activation. *Sci. Rep.* **6**, 37580; doi: 10.1038/srep37580 (2016).

**Publisher's note:** Springer Nature remains neutral with regard to jurisdictional claims in published maps and institutional affiliations.
